# Around the Hospitals

**Published:** 1893-08-12

**Authors:** 


### AROUND THE HOSPITALS.

Notice to the Managers of Institutions.—Special space is now reserved for the insertion of notices of hospital meetings and festivals. The Editor requests that all notices may reach The Hospital Office, 428, Strand, at least one week before the date of each meeting.

**The North London Hospital for Consumption.**—Since the last annual report of this institution the premises have been considerably extended. The central offices and out-patients' department are now in Fitzroy Square. The new central block, containing entrance hall, dining-room for patients, and accommodation for nurses, was opened in July. Thus the hospital has launched forth on an enlarged sphere of usefulness, and more fully equipped than before. It is reassuring for those who lament the want of funds which prevent them moving sick friends far from the smoke of London, to read the confident assertion contained in the annual report of the Hampstead Hospital. It runs: "Nowhere else (speaking of London) are more satisfactory results achieved." From the hospital itself, of a total number of in-patients amounting to 371, no less than 310 were discharged improved in health. The deaths only give a mortality of 7 27 per cent. We notice a falling-off in subscriptions. The hospital has been more fortunate in respect to both donations and legacies, which brought the receipts of 1892 to a larger total than the previous year. The principal change in the staff has been the election of a new Matron (Miss Elphick), in place of Miss Blaxland, retired.

**Charing Cross Hospital.**—The chief advance shown by the last annual report of this hospital is in the medical school, which is making a distinct advance in importance. The improvements made in the building have proved most beneficial, and the increase in the work has demanded the creation of a new appointment, that of pathologist and curator of the museum. The fees accruing to the hospital from this part of its work amounted to £1,014 during the past year. During this period the hospital was closed for two months; nevertheless, the number of in-patients treated was not very much smaller than in the previous year. Charing Cross Hospital is situated in so densely populated neighbourhood that its resources are severely taxed at all times, and it is an institution requiring a far larger income than is at present accorded to it, and the debt of £5,394 is a burden which should be speedily removed. Its efforts are recognised by an increasing subscription list, but donations last year fell far short of the usual average. The hospital was the fortunate recipient of some generous bequests, the largest of which amounted to £2,500, from the late Mrs. Chandless. The expenditure amounted to £13,184. The expense per patient will be probably lessened when the convalescent home, which is to be presented to the hospital by Mr. Passmore Edwards, is completed. The benefit of such an addition to the work of a hospital cannot be overestimated, and we hope that the next annual report of this institution will contain an announcement that the home is fast approaching completion.

**The Huddersfield Infirmary.**—This institution issues a satisfactory report of the past year's work. The in-patients received amount to 818, and the out-patients to 4,274. In addition to these a large number of patients have been visited in their own homes by the medical staff. The financial report bears a substantial proof that the working classes appreciate the benefits afforded by the hospital. The general public have not made any increase in their subscriptions, though there is no falling off. Six legacies have been received, amounting to £903. A large number of festivities were held in behalf of the institution, which added materially to the funds, and proved general interest and goodwill. The percentage of deaths in the hospital was commendably low.

